# Outcome of index upper gastrointestinal endoscopy in patients presenting with dysphagia in a tertiary care hospital-A 10 years review

**DOI:** 10.1186/1471-230X-7-43

**Published:** 2007-11-22

**Authors:** Nafees A Qureshi, Michael T Hallissey, John W Fielding

**Affiliations:** 1 Department of upper GI surgery, University Hopitals Birmingham NHS Foundation Trust, Edgbaston, Birmingham B15 2TH, UK

## Abstract

**Background:**

Patients with malignant tumours of the upper gastrointestinal tract tumours exhibit important alarm symptoms such as dysphagia that warrant clinical investigations. An endoscopic examination of the upper gastrointestinal tract will be required in most cases. This study evaluates the diagnostic potential of index endoscopy in a random population of patients with dysphagia.

**Methods:**

This is a retrospective analysis of prospectively collected data over 10 years. Patients with previous endoscopic evaluation or upper gastrointestinal pathology were excluded from the study. Data was analysed to see the number and frequency of abnormal findings in upper gastrointestinal tract, and their significance in relation to the presenting symptoms.

**Results:**

Total number of index endoscopies was 13, 881. 913 patients were included in the study including 465 males (age range: 17–92 years, median: 55 years) and 448 females (age range: 18–100, median: 59 years), with male to female ratio of 1.04: 1. Oesophagus was abnormal in 678 cases (74%) and biopsies were taken in 428 patients (47%). Superficial oesophagitis, Barrett's oesophagus, oesophageal cancer, and oesophageal ulcer were main histological findings. Age more than 50 years and weight loss were significant predictors of oesophageal cancer (p < 0.0001). Male gender, heartburn, epigastric pain, weight loss and vomiting were significantly related to Barrett's oesophagus. A total of 486 gastric and 56 duodenal biopsies were also taken. There were 20 cases of gastric adenocarcinoma.

**Conclusion:**

OGD is an effective initial investigation to assess patients with dysphagia, especially males above the age of 50 years. Patients may be started on treatment or referred for further investigations, for example, a barium meal in the absence of any anatomical abnormality.

## Background

Dysphagia is a Greek word and means disordered eating. Dysphagia typically refers to difficulty in eating as a result of disruption in the swallowing process. It is an important alarm symptom, especially when associated with other upper gastrointestinal (GI) symptoms like dyspepsia, chronic gastrointestinal bleeding, progressive unintentional weight loss, persistent vomiting, iron deficiency anaemia or epigastric mass. Dysphagia may be caused by a variety of upper GI conditions, ranging from benign to malignant. These conditions include neuromuscular or structural disorders causing dysmotility either in the oropharynx or oesophagus (oesophageal body, lower oesophageal sphincter or cardia). Although the true prevalence of dysphagia is not known, it is reported to be 16% to 22 % after the age of 50 years [1,2]. Often it leads to the finding of an anatomical or motility disorder of the oesophagus. As part of the alarm symptoms, dysphagia needs to be investigated on an urgent basis to establish a diagnosis early in the course of the patient's management and to rule out any ongoing serious pathology such as a neoplastic process. A detailed medical history and clinical examination is the key to rule out the more obvious causes of dysphagia, especially if these are related to the oropharynx. There are several diagnostic investigations available to evaluate dysphagia, including upper gastrointestinal radiography and endoscopy. Most patients with dysphagia referred to the surgical clinics have oesophageal causes, and therefore, an endoscopic examination of the upper GI tract (oespphago-gastro-duodenoscopy; OGD) as first line examination will be required in these cases.

In this study, we evaluated the diagnostic potential of OGD and looked at the nature and frequency of different upper GI conditions, benign and malignant, diagnosed on index OGDs in a random population of patients referred primarily with dysphagia. Significance of individual symptoms that formed the basis of referral was studied in relation to histological findings.

## Methods

This is a retrospective analysis of prospectively collected data over a period of 10 years (January 1995-January 2005) of all patients who presented to the department of upper gastro-intestinal surgery at the University Hospitals Birmingham NHS Foundation Trust (U.K) with dysphagia, with or without other symptoms of upper GI tract. The definition of dysphagia was similar amongst the investigators. This analysis as carried out after formal approval from the hospital's local Research and Development directorate. Only those patients who were referred by general practitioners or from outpatient clinics were included in the study. Data was retrieved from a computer database, which is dedicated solely for the endoscopy records and is specifically designed to store and update all the necessary clinical information relating to the patients undergoing endoscopy, for example, mode of referral, symptoms at the time of referral, past medical illnesses, endoscopy findings, details of any interventions done at the time of scoping (e.g. oesophageal dilatation or stenting), number and site of biopsies taken, provisional diagnosis, final diagnosis based on histopathology reports, and finally, all the necessary details of the follow-up. Data is entered into the database by the endoscopist at the end of the procedure. Those patients who had undergone oesophageal evaluation in the past, failed OGDs, or who had a history of previous upper GI pathology or surgical procedure were excluded from the study. Data was analysed by Fisher's exact test, using SPSS as statistical software to see the number and frequency of abnormal findings in oesophagus as well as other parts of the upper GI tract and their significance in relation to the symptoms for which the endoscopy was performed. Oesophageal cancer, Barrett's metaplasia, stricture, gastric cancer and gastric intestinal metaplasia were regarded as major pathologies.

## Results

Total number of index endoscopies was 13, 881. A total of 913 patients with dysphagia were included in the study including 465 males (age range: 17–92 years, median: 64 years) and 448 females (18–100 years, median: 67 years), with male to female ratio of 1.04: 1. Out of these 913 patients, 752 were above the age of 50 years and 161 were up to or below 50 years. Only one endoscopy failed because of technical reasons. Dysphagia was a sole symptom in 388 cases; in rest of the cases, it was associated with other upper GI symptoms (Table 1). Grossly, oesophagus was abnormal on endoscopy in 678 cases (74%). Oesophageal biopsies were taken in 428 patients (47%); findings in the remaining 250 patients were convincingly those of grade 1 oesophagitis and so no biopsies were taken. Various oesophageal conditions diagnosed on histology are shown in Table 2. Correlation of symptoms and patient factors with various oesophageal diagnoses is shown in Table 3. There was no oesophageal cancer below the age of 40 years. Dysphagia associated with other upper GI symptoms (but not dysphagia in isolation), age more than 50 years, male gender, weight loss and heartburn were significantly associated with oesophageal cancer. Male gender was significant risk factor for Barrett's oesophagus (BA). Only 2% of all the oesophageal biopsies were reported as normal. Superficial oesophagitis, Barrett's oesophagus, oesophageal cancer, and oesophageal ulcer were main histo-pathological findings. Superficial oesophagitis was the most common oesophageal diagnosis (182/913) with a prevalence of 19.8%. Prevalence of oesophageal cancers (OC) and Barrett's oesophagus (BA) was 8.1% and 13.8% respectively. Oesophageal cancers included 52 cases of adenocarcioma (70.2%), 17 squamous cell carcinomae (23.1%), 1 signet cell carcinoma (1.35%), 2 anaplastic cancers (2.70%) and 2 patients had undifferentiated tumours with neuro-endocrine differentiation (2.70%).

**Table 1:** Symptoms in addition to dysphagia

**Additional symptoms:**	**No. Of patients (%):**
Heartburns	246 (27%)
Epigastric pain	203(22%)
Weight loss	135 (15%)
Vomiting	136 (15%)
Flatulence	34 (3.4%)
Anaemia	33 (3.6%)
Malaena	6 (0.7%)
Upper abdominal mass	4 (0.4%)

**Table 2:** Oesophageal diagnoses on histology

**Condition:**	**No. Of cases:**	**% Of all cases:**
Superficial Oesophagitis	182	19.9
Barrett's oesophagus	126	13.8
Oesophageal cancers	74	8.1
Oesophageal ulcers	35	3.8

**Table 3:** Correlation of symptoms with major oesophageal disorders

	**Oesophageal cancer**		**Barrett's oesophagus**	
**VARIABLE**	**OR (95% CI)**	**P value**	**OR (95% CI)**	**P value**

Male Gender	1.868 (1.138–3.068)	0.0149*	1.497 (1.021 to 2.194)	0.0437
Age > 50	3.152 (1.251 to 7.945)	0.0100*	0.9522 (0.5845 to 1.551)	0.8026
Dysphagia associated with other symptoms	1.707 (1.024 to 2.846)	0.0491*	0.8466 (0.5801 to 1.235)	0.4376
Anaemia	1.139 (0.3392 to 3.827)	0.5100	0.6504 (0.2183 to 1.938)	0.6076
Epigastric pain	0.7280 (0.3915 to 1.354)	0.1962	0.8960 (0.5640 to 1.424)	0.7294
Heartburn	0.5543 (0.2989 to 1.028)	0.0355*	1.099 (0.7238 to 1.669)	0.6660
Vomiting	0.8842 (0.4421 to 1.768)	0.8651	0.7438 (0.4194 to 1.319)	0.3477
Weight loss	3.148 (1.860 to 5.328)	< 0.0001*	0.6880 (0.3819 to 1.239)	0.2786

* Significant

A total of 486 gastric and 56 duodenal biopsies were also taken due to abnormalities found in the stomach and duodenum. This included 20 cases of gastric adenocarcinoma. Nineteen of these patients were above the age of 50 years but the relationship between age and gastric cancer was not significant (P = 0.2305). Results of the gastric and duodenal histology are shown in Table 4.

**Table 4:** Gastric and duodenal diagnoses on histology

**Condition:**	**No. Of cases:**	**Percentage of total cases:**
Superficial gastritis	169	18.5
Intestinal metaplasia	73	7.99
Helicobacter gastritis	141	15.5
Foveolar hyperplasia	79	8.7
Gastric cancer	20	2.2
Duodenitis	12	1.3

## Discussion

Malignant tumours of the upper gastrointestinal tract (oesophagus and stomach) account for 13,300 deaths and approximately 16,600 new cases each year in the UK [3]. These tumours usually have a long natural history and may present at a fairly advanced stage. Nevertheless, patients with these tumours exhibit important alarm symptoms, for example, dysphagia, dyspepsia, chronic gastrointestinal bleeding, progressive unintentional weight loss, progressive difficulty in swallowing, persistent vomiting, iron deficiency anaemia or epigastric mass that warrant further clinical investigations. Urgent specialist referral within two weeks is required for endoscopic investigation for patients with dysphagia.

Upper GI endoscopy is an important tool in the initial investigation of dysphagia [4]. Although widely used, there are few reports about the diagnostic potential of OGD in a random group of patients with dysphagia. There are, however, studies which have reported on the usefulness of OGD in patients with HIV, bone marrow transplantation and connective tissue disorders who suffer from dysphagia [5-7]. Our study is an effort to find out different conditions that can present as dysphagia in a random population of patients and relationship between the presenting symptoms and endoscopic as well as histological findings.

Our data suggests that dysphagia and the presence of other alarm symptoms are good predictors of the presence of major endoscopic findings in the upper gastrointestinal tract and there is a good chance of finding important upper gastrointestinal conditions such as ulcer disease, cancer, and strictures. Although previous studies show age 40 years and above to be significant for the development of oesophageal cancer [8], there were only 5 cancers between the age of 41–50 years in our study, with no statistical significance. No cancer was found below the age of 40 years. Male gender was significant risk factors for Barrett's metaplasia. Anaemia was not significantly associated with any major oesophageal pathology.

Although majority of the patients with dysphagia and related symptoms are more likely to suffer from benign conditions like superficial oesophagitis, chances of having Barrett's oesophagus or even cancer as underlying pathology are not uncommon.

This study recommends completion of OGD and a detailed examination of the rest of the upper GI tract in all cases with dysphagia as there is a significant chance of finding gastric disorders in theses patients. We found 20 cases of gastric adenocarcinoma in our study population; cancer was present in the region of cardia in 11, fundus and body in 4 and antral region in 5 cases. Majority of these patients were above the age of 50 years but the relationship between age and gastric cancer was not significant (P = 0.2305).

## Conclusion

Upper GI endoscopy is an effective and appropriate initial investigation to assess patients with dysphagia, especially in males above the age of 50 years with or without additional symptoms like weight loss, heartburn and vomiting. This study would recommend taking multiple biopsies in any abnormal looking upper GI mucosa to reach a safe and definite diagnosis. OGD will help in making a provisional diagnosis in most cases and biopsies can be taken from the suspected lesions to get histological evidence. At the same time, patients may be started on treatment for benign but potentially harmful conditions like gastro-oesophageal reflux disease. Patients may also be referred for further investigations in the absence of any anatomical abnormality in cases of dysphagia. Radiological studies such as fluoroscopy or Barium swallow/meal may explain any motility disorders in cases of normal endoscopy findings of the upper gastrointestinal tract.

## Competing interests

The author(s) declare that they have no competing interests.

## Authors' contributions

NAQ carried out the study and authored the initial and final draft of the paper. MTH reviewed the draft and suggested vital changes in the initial draft. JF conceived the study and reviewed the paper for final approval. All authors read and approved the final manuscript.

## Pre-publication history

The pre-publication history for this paper can be accessed here:


